# The bionomics of *Anopheles arabiensis* and *Anopheles funestus* inside local houses and their implications for vector control strategies in areas with high coverage of insecticide-treated nets in South-eastern Tanzania

**DOI:** 10.1371/journal.pone.0295482

**Published:** 2024-12-05

**Authors:** Alex J. Limwagu, Betwel J. Msugupakulya, Halfan S. Ngowo, Yohana A. Mwalugelo, Masoud S. Kilalangongono, Faraji A. Samli, Said K. Abbasi, Fredros O. Okumu, Billy E. Ngasala, Issa N. Lyimo

**Affiliations:** 1 Environmental Health and Ecological Sciences Department, Ifakara Health Institute, Morogoro, Tanzania; 2 Parasitology and Medical Entomology Department, Muhimbili University of Health and Allied Sciences, Dar es Salaam, Tanzania; 3 Department of Vector Biology, Liverpool School of Tropical Medicine, Liverpool, United Kingdom; 4 Department of Biomedical Sciences, Jaramogi Oginga Odinga University of Science and Technology, Bondo, Kenya; 5 School of Life Science and Bioengineering, Nelson Mandela Africa Institution of Science & Technology, Arusha, Tanzania; 6 School of Biodiversity, One Health, and Veterinary Medicine, G128QQ, University of Glasgow, Glasgow, United Kingdom; University of Ibadan Faculty of Science, NIGERIA

## Abstract

**Background:**

Residual malaria transmissions in Africa may be associated with improved coverage of insecticide-treated nets, house features, and livestock husbandry. These human-land use activities may drive the ecology and behaviour of malaria vectors which sustain residual malaria transmission. This study was conducted to assess changes in the ecology and behaviour of *Anopheles funestus* and *Anopheles arabiensis* in villages with high coverage of insecticide-treated nets to guide the selection of complementary vector control strategies against residual malaria transmission.

**Methods:**

Mosquitoes were collected using a CDC-light trap, miniaturized double net trap, and Prokopack aspirator from 222 households in three villages (Ebuyu, Chirombora, and Mzelezi) within Kilombero Valley. *Anopheles* mosquitoes were morphologically identified to their physiological status and species-complex levels. A sub-sample of *Anopheles* mosquitoes was exposed to laboratory analyses of sibling species, host preference, and sporozoite rates. Additionally, the local houses were geo-referenced using Global Positioning Systems (GPS) devise, and house features were recorded and associated with vector abundance.

**Results:**

The population of *An*. *funestus* s.s was abundant with high *Plasmodium* sporozoite rates inside houses compared to *An*. *arabiensis*. However, these vector species equally blood-fed on humans inside houses, but they also flexibly mixed human and animal blood meal. Fewer *An*. *funestus* were caught in houses with metal- than grass roofs and houses with and without animals. Contrastingly, fewer *An*. *arabiensis* were caught from houses with screened eaves compared to houses with open eaves.

**Conclusions:**

This study confirms that *An*. *funestus* dominates residual malaria transmission over *An*. *arabiensis*. These vector species exhibit anthropophily and opportunistic blood-feeding behaviour in areas with high coverage of insecticide-treated nets, but they numerically respond differently to local house improvements. These results imply that integrating mosquito-proof houses, improved insecticide-treated nets, and livestock-based interventions could effectively reduce and eventually eliminate residual malaria transmission.

## Background

Despite being preventable and curable, malaria continues to pose a persistent global public health challenge which causes 608,000 deaths and 249 million cases worldwide in 2022, a decrease of 11,000 deaths, and an increase of 5 million cases compared to 2021 [1, 2]. The majority of such global malaria burden (i.e. >94%) occurs in sub-Saharan Africa, particularly in children under five years, adolescent girls and pregnant women [3–6]. The protozoan parasites of genus *Plasmodium* with five known species such as *P*. *falciparum*, *P*. *vivax*, *P*. *ovale*, *P*. *malariae*, *and P*. *knowlesi* are responsible for causing malaria burden in humans [7]. *Plasmodium falciparum* is predominantly the deadliest malaria parasite carrying 96% of global malaria infections, particularly prevalent in Africa [8, 9]. Whereas the *Plasmodium vivax* is the second most important malaria parasite causing almost 2% of global malaria infections, and it is most prevalent/dominant in Southeast Asia and America [8, 10]. The other *Plasmodium* species are less prevalent and rarely cause deaths including *P*. *malariae*, and *P*. *ovale* in Africa [11–13], and Southeast Asia [14, 15], and *P*. *knowlesi* which causes malaria in humans and monkeys mainly in Southeast Asia [16, 17]. Malaria parasites are transmitted between humans through bites of *Anopheles* mosquitoes, with *An*. *gambiae* s.s, *An*. *arabiensis*, *An*. *coluzii*, and *An*. *funestus* s.s being major African malaria vectors [18, 19]. The variations in ecology and blood feeding behaivour of these major malaria vectors drive malaria transmission intensity [20, 21].

Major malaria vector control strategies in Africa include Insecticide Treated Nets (ITNs) and Indoor Residual Spraying (IRS) [22]. The effectiveness of these intervention exploits the behaviour of malaria vectors inside houses [23]. The scaling-up of these indoor-based interventions has successfully reduced the burden of malaria in Africa from 2000 to 2017 [24, 25]. These interventions effectively target malaria vectors such as *An*. *gambiae* s.s and *An*. *funestus* s.s which blood feed entirely on humans (anthropophily), inside houses (endophagy), and rests on the wall inside houses (endophily) [26]. However, the progress in reducing the malaria burden has stalled/plateaued with malaria control responses remaining at a cross-road/off track from 2017 to present due to ongoing persistent malaria transmission even in areas with high/absolute coverage of ITNs and IRS, which is known as residual malaria transmission [1, 27]. Malaria vectors evade ITNs and IRS through increased physiological insecticide resistance, behavioural changes, and vector species composition changes [28–31]. These vectors sustain residual malaria transmission in African countries which threatens to achieve the global technical strategy (GTS) goal of eliminating malaria by 2030. Therefore, a clear understanding of the bionomics of primary malaria vectors in areas with high coverage of ITNs is urgently needed to inform the selection of effective vector control intervention packages to complement ITNs and IRS in reducing residual malaria transmission and eventually reaching the global technical strategy goal of malaria elimination in Africa.

The residual malaria transmission may have several drivers in rural villages of African countries. The high coverage of ITNs and IRS inside houses, along with the application of insecticides on livestock and crop production have significantly contributed to increased selection pressure on endophilic and anthropophilic malaria vector populations. The development of widespread physiological insecticide resistance enables these vectors to survive the contact with insecticides on nets and walls to sustain malaria transmission inside houses [32, 33]: Notably, the insecticide resistance mechanisms such as target site insensitivity were observed in *An*. *arabiensis*, *An*. *funestus* and *An*. *gambiae* s.s [34–37], and metabolic insecticide resistance found in *An*. *funestus* and *An*. *arabiensis* [34, 36–41]. Malaria vectors can also adapt to changing environments of house designs, interventions ITNs/IRS, and the presence of alternative host species through behavioural modifications. These vector behavioural changes enable them to evade ITNs and IRS inside houses through changing biting time (e.g. from night to early morning/evening, daytime, and late night) when people are out of bed nets [33, 42–45], biting and resting location (i.e. from indoors to outdoors) [42, 44–50], and host choice (i.e. from feeding on humans (anthropophilic) to livestock (zoophily or opportunistic/plastic) [42, 48–56] Additionally, the ITNs and IRS could also induce numerical changes in vector populations leading to changes in vector species composition due to disproportionately reduced abundance of one vector species. High coverage of ITNs/IRS have significantly reduced the abundance of *An*. *gambiae* s.s or *An*. *funestus* s.s to undetectable levels inside and outside houses in most areas, leaving behaviourally resilient *An*. *arabiensis* sustaining residual malaria transmission in African countries including Tanzania [50, 52, 57–60].

Some of the physical house features where ITNS and IRS are applied in rural villages may also drive the exposure of humans to malaria vectors. For example, the house features include eaves, windows, doors, and keeping of livestock which are associated with mosquito entry and the abundance of mosquitoes inside houses. The previous studies on the association of households with livestock and the risks of exposing humans to malaria vectors in rural villages generated mixed results: 1) livestock attracted more malaria vectors to inside houses, provided an alternative blood meal source for reproduction, created more breeding habitats; consequently increased increase vector abundance and thus increases malaria risks to humans [53, 61–64] 2) livestock diverted malaria vectors away from humans, and reduced vector abundance inside houses, human mosquito biting rates/preferences, and thus reduced malaria risks to humans [53, 65–67]; and 3) no effect livestock on abundance of vector inside houses and malaria risks- it was as good as not having livestock close to households [68, 69]. These studies suggest that the effect of keeping livestock in/near houses on vector abundance and risk of malaria to humans could be linked with the type of local vector species, their ecology, and behaviour; livestock type and density, the distance between livestock and house, and local house features. Furthermore, previous studies demonstrated that malaria risk in rural villages was higher in poor house designs with open eaves, windows, mud walls, and thatched roofs (i.e., owned by the majority of residents) than those improved households with closed eaves, and metal roofs (i.e., mosquito proof houses) [70–72]. Such mosquito-proof houses may reduce the abundance of mosquitoes inside, but increase outdoor biting malaria vectors similar to ITNs and IRS inside houses. The impact of household features such as ITNs/IRS, livestock husbandry, and house improvements on the abundance, species composition, and behavior of primary malaria vectors is still a complex issue. further investigations these household features on malaria vectors bionomics in different ecological settings are required to inform the selection of complementary vector control strategies to ITNs.

This study aimed to evaluate the variations in the bionomics of primary malaria vectors inside houses and their implications for selecting effective complementary vector control strategies in regions with high ITN coverage (approximately 77.0%) within the Kilombero Valley in southeastern Tanzania. The specific aims of this study were therefore: 1) to determine the variation in abundance and *Plasmodium falciparum* infections between *An*. *arabiensis* and *An*. *funestus*, 2) to assess variation in blood feeding preferences between *An*. *arabiensis* and *An*. *funestus*, 3) to evaluate the impact of house improvements on the abundance of *Anopheles arabiensis* and *Anopheles funestus* inside houses.

## Materials and methods

### Study area

**Fig 1:**
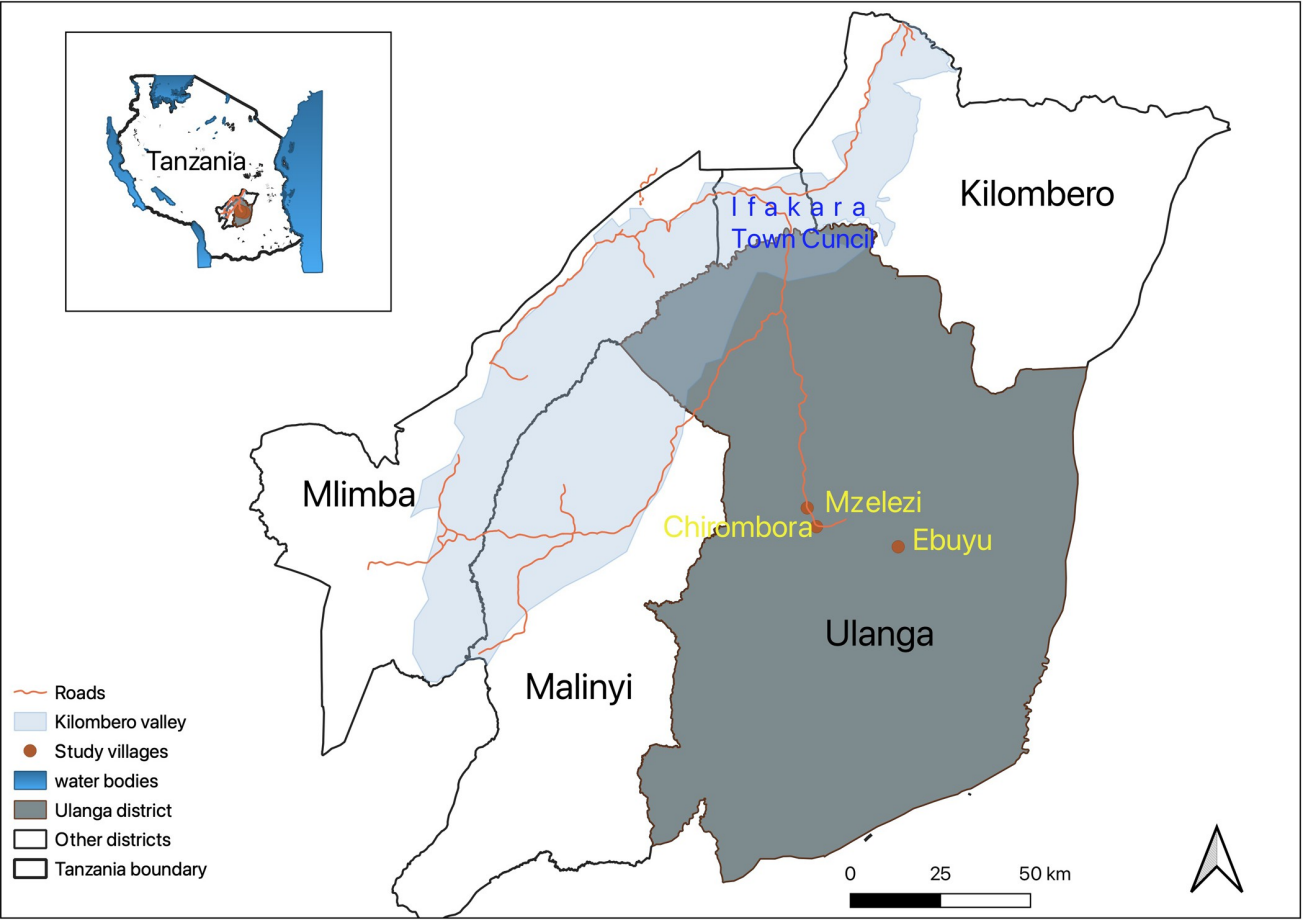
Map of the study area showing three villages in Ulanga district, south-eastern Tanzania.

The cross-sectional entomological survey was conducted in three different villages in Ulanga district within Kilombero Valley located in the south-eastern region of Tanzania (Fig 1). Mosquitoes were collected from three villages: Ebuyu (-8.979 S, 36.760 E), Chirombola (-8.926 S, 36.753 E), and Mzelezi (-8.898 S, 36.735 E). Average annual rainfall ranged between 1200 and 1800 mm, and the mean annual temperature was 20–32°C, as reported by Ngowo et al. in 2017 [73]. Most residents in these villages practice subsistence agriculture including rice and maize cultivation; livestock husbandry and fishing as their primary economic activities. The typical local housing structures in this valley have the following features: clay brick walls, open eaves (the space between the roof and walls), and open windows. These human land use activities create favorable environments for breeding, feeding, resting, and survival of main malaria vectors that facilitate high mosquito densities throughout the year. Currently, the major malaria vectors in this valley that sustain malaria transmission include *An*. *arabiensis* and *An*. *funestus* [54, 74, 75]. These vectors mainly transmit *Plasmodium falciparum* which causes most of the malaria cases in the area, with *An*. *funestus* mediating >90% of malaria transmission in this valley [74, 76]. Another human land use activity in this valley is the use of ITNs against malaria vectors which has now reached approximately 77% of ITN coverage, and 63.3% of ITN usage.

### Mosquito sampling/collections from indoors and outdoors

The cross-sectional entomological survey was conducted from March 2022 to July 2022, a period that covered the rainy/wet (March ‐ May) and dry (June ‐ Sept) seasons. The abundance of malaria vectors such as *An*. *arabiensis* and *An*. *funestus* in the villages peaks during wet seasons, but it decreases during dry seasons. However, the abundance of *An*. *funestus* persists in some villages due to their preference for permanent/semi-permanent breeding habitats which exist throughout the year. Adult mosquitoes were collected from a total of 222 households in three different villages (i.e., 74 households/village x 3 villages = 222 households) as described previously [77]. Each trap design (i.e., CDC light traps and DN-Mini traps) was randomly assigned to 36 households and 1 household for each trap was used as a sentinel household (i.e. 36 households/trap design x 2 trap designs + 1 sentinel household/trap design x 2 trap designs = 74 households) as described previously [77]. The host-seeking mosquitoes were collected indoors and outdoors by using a double net trap (DN-mini trap) as described previously [78], but it was only from indoors using the CDC light trap as described previously [79]. The resting mosquitoes were also collected indoors and outdoors from sentinel households using a Prokopack aspirator as described elsewhere [80]. The Prokopack aspirator was introduced in houses with the DN-Mini trap and CDC-light trap to collect resting mosquitoes from both indoors and outdoors. These resting mosquitoes collected in Prokopack aspirators were used to understand the species composition and blood feeding behavior of mosquitoes that were not actively seeking hosts at the time of collection. However, the collection of host-seeking mosquitoes by DN-Mini trap and CDC-light trap aimed to capture mosquito host seeking behavior and species diversity within the area. Host-seeking mosquito collections were performed from 6 p.m. to 6 a.m., but resting mosquito collections were conducted from 6.45 a.m. to 7.00 a.m. The CDC light trap with a lid was hung about 1–1.5 m above the ground under an occupied bed net, but the DN-Mini trap was allocated in the sitting room, with male volunteers aged between 18years to 45 years inside acting as bait and collecting mosquitoes after every one-hour interval. A pair of volunteers in DN-Mini traps were rotated between night as described previously by Limwagu et al (2024) [77]. The resting mosquitoes were searched and collected from ceilings, under chairs, cooking pans, hung clothes, and water basins for indoor environments, they were also collected from buckets, clay pots, and car tires around 5metres from sentinel households for outdoor environments. Mosquitoes were collected from each household for three days a week for four months. Each time host-seeking mosquito collections from random households, 2 households were covered per day for 18 days per trap design. The resting mosquitoes were repeatedly collected from sentinel households at a rate of once per week for four months (1 day/week x 4 weeks/month x 4 months = 16 days). Additionally, all the sampled houses were also characterized through the collection of the variables including eave space status, roof type, wall type, window status, presence of animals and poultry inside and outside (i.e., around 100 meters) from each sampled houses, latrine location inside or outside houses. Geographical positioning system (Garming Extrex 20, GPS) device was used to collect GPS coordinates of each sampled house were also collected during household characterization.

### Detection of malaria vector sibling species and their *Plasmodium* infection rates

Female *Anophel*es mosquitoes collected from inside and outside houses using three different traps were killed each morning using petroleum or alcohol fumes. These mosquitoes were morphologically sorted by taxa and physiological status. While the taxa were recorded as *Anopheles gambiae* complex or *An*. *funestus* group, the abdominal status of each female *Anopheles* mosquitoes was recorded as unfed, partly-blood fed, fully-blood fed, semi-gravid, or gravid. A sub-sample of *An*. *gambiae* complex and *An*. *funestus* group was packed individually in 1.5 ml microcentrifuge (Eppendorf®) tubes filled with silica gel and a piece of cotton wool. These mosquito samples were submitted to the Ifakara Health Institute molecular laboratory for sibling species identification using multiplex polymerase chain reaction (PCR). The sibling species of *An*. *gambiae* complex was identified using Scott’s protocol [81], but the members of *An*. *funestus* group were identified using Koekemoer’s protocol [82]. Additionally, a sub-sample of mosquitoes, consisting of whole bodies with intact body parts, was pooled, with no more than 10 individuals per species, and prepared for Circumsporozoite Protein Enzyme-Linked Immunosorbent Assay (CSP-ELISA). The head and thorax of mosquitoes in these pools were separated from the abdomen and tested for the presence of *Plasmodium falciparum* circum-sporozoite protein (*Pf* CSP) in their salivary glands using the enzyme-linked immunosorbent assay (ELISA) method [82]. To prevent detection of false positives, the ELISA lysates were boiled for 10minutes at 100°C to completely eliminate heat labile antigens of non-*Plasmodium falciparum*.

### Detection of blood meal sources in malaria vectors

The blood meal content of *Anopheline* mosquitoes that were collected from all traps which were placed indoors and outdoors was analyzed using enzyme-linked immunosorbent assay (ELISA). The abdomens of all blood-fed malaria vectors collected were examined to determine their blood meal sources such as from one or a mixture of these host species: humans, goats, dogs, chickens, and bovines. Monoclonal antibodies (Antisera) against humans, cattle, goats, chickens, and dogs immunoglobulin G [IgG] identifiers (KPL, Gaithersburg, MD, USA) were utilized to identify blood meal sources from these different host species following the procedure described by Beier et al., [83].

### Assessing the effects of household characteristics on malaria vector density

Mosquitoes were collected from both indoors and outdoors using three different traps (CDC light traps, DN Mini traps, and Prokopack aspirators). The participants also recorded various house characteristics. These details included the house type, construction materials, presence or absence of animals inside and outside around 100 meters from the houses. The overall condition of the house, encompassing factors like the presence of eave spaces, and the status of window and door screening. Additionally, precise coordinates of the house location were collected using a GPS device and concurrently saved in a data collection form. Such data collection form contained more information than what was initially stored in the GPS device. Finally, the effects of these household characteristics on malaria vector density were evaluated.

### Data analysis

All data were analyzed using open-source statistical software, R program language version 4.2.11 [84]. The descriptive analysis was performed to summarize data on entomological indices including mosquito abundance, species composition, blood meal sources, and sporozoite rates using frequencies, mean, and proportions. Furthermore, the generalized linear mixed model directly incorporated negative binomial and zero-inflated functions using template model builder (GlmmTMB) was used to analyze mosquito count data [84]. Mosquito counts were modeled as response variables, while house characteristics were modeled as fixed terms. Additionally, the household ID and sampling date were included in the model as random terms to account for any unexplained variations between houses and pseudo-replicates. Significance levels were considered at p<0.05.

### Ethical considerations

The study was conducted following the principles of the Declaration of Helsinki. Ethical approval for conducting this study was obtained from the Muhimbili University of Health and Allied Sciences Review Board (MUHAS-REC-12-2021-910). While the permission to publish the work was approved by NIMR (Ref No. BD.242/437/01A/17). Additionally, the permission to conduct this study in three villages within Ulanga district was also secured through the approval of the District Medical Officer of Ulanga district and the cooperation of local government authorities in the chosen villages. Before commencing the execution of the study, meetings were convened with local government leaders, and community members including the head of household to elucidate the objectives, methodologies, risks, and benefits of the study. The research team diligently obtained both verbal and written informed consent from all individual residents of households and the human volunteers engaged in mosquito collection in the mosquito traps. Participants were provided with detailed information regarding the potential benefits and risks associated with their participation, and their voluntary involvement was emphasized and respected. Participants were also assured of their right to withdraw from the study at any point if they wished to do so, without asking for any reasons for facing adverse consequences. Participants were also guaranteed confidentiality and ensured the anonymity of all participants.

## Results

### Mosquito abundance and their composition

A total of 19851 female mosquitoes were collected indoors and outdoors from March to July 2022 using the CDC-light trap, DN-Mini trap, and Prokopack aspirator. Among all host-seeking mosquitoes, the CDC-light trap captured 45.3% (n = 9008), the DN-Mini trap captured 39.5% (n = 7851) from indoors, but the DN-Mini trap also captured 4.4% (n = 881) from outdoors. Additionally, Prokopack aspirators captured resting mosquitoes at a rate of 8.8% (n = 1747) from indoors, and 1.8% (n = 364) from outdoors. *Anopheles* mosquitoes collected in traps: *An*. *funestus* group. were 6.14% (n = 1219), *An*. *gambiae* s.l. were 1.57% (n = 313), and *An*. *coustani* was 0.05% (n = 10). On the other hand, *Culex* species were the majority of mosquitoes collected in traps at a frequency of 92.07% (n = 18268), *Mansonia* were 0.14% (n = 29), and *Aedes* species were 0.01% (n = 2).

### Malaria vector species composition

A sub-sample of 487 female *Anopheles* mosquitoes were submitted to the molecular laboratory for sibling species identification. Out of these mosquitoes, 419 individuals were morphologically identified as *An*. *funestus* group, and 68 individual mosquitoes as *An*. *gambiae* complex. When the PCR was performed on *Anopheles* mosquito samples, the amplified samples revealed sibling species of *An*. *funestus* group were *An*. *funestus* s.s (87.59%, n = 367) and *An*. *rivulorum* (0.24%, n = 1), while unamplified samples were 12.17% (n = 51). The amplified sibling species of *An*. *gambiae* s.l were *An*. *arabiensis* (88.24%, n = 60), and *An*. *gambiae* s.s (5.88%, n = 4), while the unamplified samples were 5.88% (n = 4).

### *Plasmodium* sporozoite rate malaria vectors

A sub-sample of 487 *Anopheles* mosquitoes collected using CDC light traps and DN-Mini traps were further analyzed in the molecular laboratory to determine their *Plasmodium falciparum* infection rates through exposure to ELISA. Out of these *Anopheles* mosquitoes, 14.0% (n = 68) were morphologically identified as *An*. *gambiae* s.l and 86.0% (n = 419) were identified as *An*. *funestus*. The *Plasmodium falciparum* sporozoites rate was 1.4% (n = 1) in *An*. *arabiensis* and 3.1% (n = 13) in *An*. *funestus* group. Sporozoite infections in *An*. *arabiensis* were detected in mosquitoes caught using DN-Mini traps placed indoors in Ebuyu village during the dry season. For *An*. *funestus*, all 13 sporozoite infected mosquitoes were detected in samples collected from indoors in three different villages. In Chirombora village, one mosquito from a CDC-light trap and two mosquitoes from DN-Mini traps. In Ebuyu village, one mosquito from CDC-light trap and four from DN-Mini traps. In Mzelezi village, two mosquitoes were from CDC-light traps and three mosquitoes were from DN-Mini traps. Regarding seasonality, nearly all infections in these mosquitoes were detected during the wet season, except for three *An*. *funestus* mosquitoes captured in the dry season.

### Blood meal sources in malaria vectors

**Fig 2:**
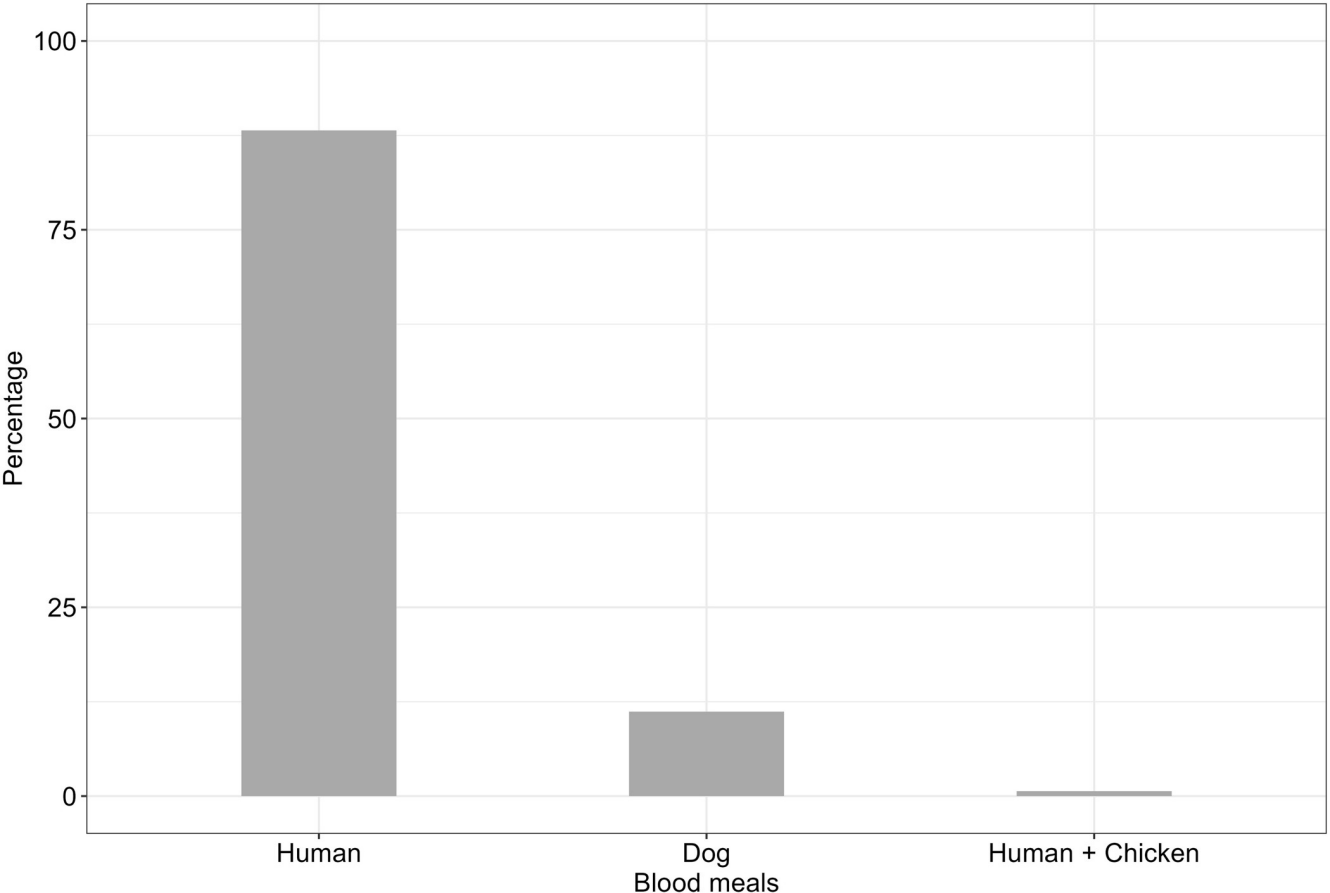
Blood meal contents of *An*. *funestus* from different host species identified by the ELISA method from the sample collected in three different villages.

**Fig 3:**
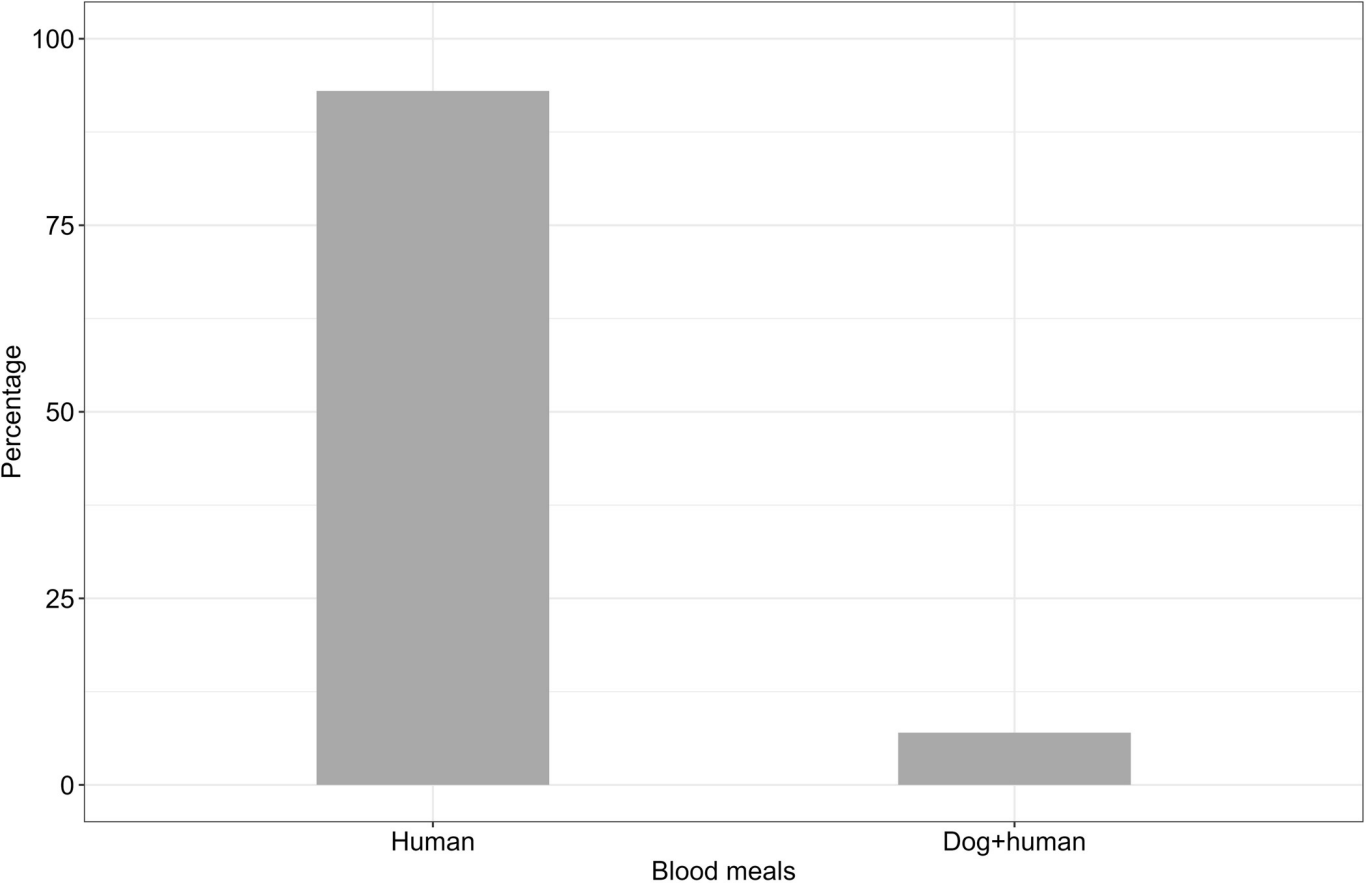
Blood meal contents of *An*. *arabiensis* from different host species identified by the ELISA method from the sample collected in three different villages.

A total of 449 female Anopheline mosquitoes collected using CDC light traps, DN-Mini traps, and Prokopack aspirators were morphologically observed to have ingested a blood meal in their abdomen. The abdominal samples with blood contents were exposed to ELISA to detect blood meal sources. Out of 449 Anopheline mosquitoes, 420 individuals were *An*. *funestus* and 29 were *An*. *arabiensis*. Out of 449 female *Anopheles* mosquitoes submitted to molecular laboratory for blood meal-ELISA analysis, mosquitoes with blood meal source identified were 37.2%, (n = 167), and unidentified were 62.8%, (n = 282). Among 167 confirmed blood fed *Anopheles* mosquitoes: *An*. *funestus* were 152, and *An*. *arabiensis* were 15. The predominant host blood antigens detected in *An*. *funestus* were humans (88.2%), followed by dogs (11.2%), while *An*. *arabiensis* had 93.3% for human blood meal (Figs 2 and 3). Additionally, these vector species were observed to mix their blood meals: mixture of blood from humans and dogs (6.7%) for *An*. *arabiensis*; and mixture of blood from humans and chickens (0.6%) for *An*. *funestus* (Figs 2 and 3).

Mosquitoes predominantly fed on humans, with 136 indoor and 11 outdoor. Feeding on dogs and mixed hosts (Dog/Human, Human/Chicken) as observed only indoors. During the wet season, mosquitoes showed a broader range of host feeding, with higher counts for both dog and human blood meals compared to the dry season Table 1.

**Table 1:** Number of blood meal sources of malaria vectors across different hosts in various location (indoors and outdoors) and seasonality (wet and dry).

Variables		Host	Total
**Location**	Indoor	Dog	17
		Dog/Human	1
		Human	136
		Human/Chicken	1
	Outdoor	Dog	0
		Dog/Human	0
		Human	11
		Human/Chicken	0
**Season**	Dry	Dog	0
		Dog/Human	0
		Human	34
		Human/Chicken	0
	Wet	Dog	17
		Dog/Human	1
		Human	113
		Human/Chicken	1

### Effects of household characteristics on vector density

Mosquito catches from inside houses were influenced by the house characteristics. Notably, fewer *An*. *funestus* were caught from inside houses with metal roofs compared to houses with grass roofs (RR = 0.646, p <0.05, Table 2). Similarly, fewer numbers of *An*. *funestus* were caught from inside houses with animals than houses without animals (RR = 0.672, p <0.01, Table 2). However, the number of *An*. *funestus* inside houses were similar between houses with open versus screened ceilings (RR = 0.780, p = 0.454, Table 2), open versus screened eave spaces (RR = 0.978, p = 0.864, Table 2), or bricks versus mud wall materials (RR = 0.690, p = 0.107, Table 2). Conversely, the catches of *An*. *arabiensis*. were significantly lower in houses with screened eaves compared to houses with open eaves (RR = 0.108, p < 0.01, Table 2). However, the number of *An*. *arabiensis* caught from inside houses were similar between houses with grass versus metal roofs (RR = 1.600, p = 0.587, Table 2), open versus screened ceilings (RR = 0.526, p = 0.575, Table 2), brick versus mud wall materials (RR = 0.351, p = 0.225, Table 2), and presence versus absence of animals (RR = 0.385, p = 0.254, Table 2).

**Table 2:** Effect of household characteristics on malaria vector densities inside houses.

Species	Variable (Household Characteristics)	Total number of mosquitoes indoors	Type	Mean ± 2SE	RR [95% CI]	p-values
*Anopheles funestus*	Roof Materials	271	Grass	2.24 ± 0.972	1	<0.05
		624	Metal	2.10 ± 0.276	0.646 [0.418, 0.998]	
	Ceiling	269	No	2.12 ± 0.272	1	= 0.454
		2	Yes	2.00 ± 1.20	0.780 [0.407, 1.495]	
	Eaves	858	Open	2.02 ± 0.314	1	= 0.864
		324	Screened	2.39 ± 0.498	0.978 [0.761, 1.258]	
	Wall Materials	201	Brick	2.15 ± 0.285	1	= 0.107
		68	Mud	1.76 ± 0.717	0.690 [0.439, 1.083]	
	Animal (100)	170	No	2.47 ± 0.402	1	<0.01
		31	Yes	1.71 ± 0.331	0.672 [0.534, 0.847]	
*Anopheles arabiensis*	Roof Materials	81	Grass	0.206 ± 0.199	1	= 0.587
		166	Metal	0.203 ± 0.083	1.600 [0.294, 8.715]	
	Ceiling	81	Open	0.203 ± 0.079	1	= 0.575
		0	Screened	0.2 ± 0.392	0.526 [0.055, 4.993]	
	Eaves	247	Open	0.231 ± 0.096	1	<0.01
		66	Screened	0.120 ± 0.108	0.108 [0.022, 0.536]	
	Wall Materials	71	Brick	0.206 ± 0.084	1	= 0.225
		10	Mud	0.176 ± 0.154	0.351 [0.065, 1.899]	
	Animal (100)	66	No	0.115 ± 0.080	1	= 0.254
		5	Yes	0.301 ± 0.135	0.385 [0.074, 1.986]	

## Discussion

This study demonstrates that *Anopheles funestus* and *Anopheles arabiensis* are the main malaria vectors in Kilombero Valley. The former vector species contributing greatly in sustaining residual malaria transmission. *Anopheles funestus* has higher abundance and *Plasmodium falciparum* sporozoite rates compared to *An*. *arabiensis*. However, *An*. *funestus* and *An*. *arabiensis* expressed highest degree (i.e., >85%) of blood feeding on humans (anthrophily) inside houses (endophagy). These vector species can also flexibly feed on animals or mix blood meals from humans and animals, but *An*. *arabiensis* have high ability of mixing host species than *An*. *funestus*. The abundance of *An*. *funestus* inside houses was significantly reduced in houses with metal than grass roofing materials, and houses with the presence than the absence of animals. Such abundance of *An*. *funestus* was never affected in houses with brick wall materials or screening eave spaces and ceilings. Contrastingly, the abundance of *An*. *arabiensis* inside houses was reduced through screening eaves, but it was never reduced through house improvements using brick wall materials, metal roofs, screening ceilings, or the presence of animals outside houses. Overall, the population of *An*. *funestus* mediates more residual malaria transmission than *An*. *arabiensis*, but some of local house features may reduce the potential of these vectors to transmit malaria in rural villages of Kilombero Valley.

Our study found that *An*. *funestus* has a high abundance and *Plasmodium falciparum* sporozoite rates than *An*. *arabiensis* that enable it to sustain residual malaria transmission in three villages within Kilombero Valley. Possibly, the increasing abundance of *An*. *funestus* in the study area could be linked with its greater life-expectancy/daily survival probability/parity rate, and high intensity of pyrethroid resistance (e.g. metabolic resistance) against ITNs [39, 85]. These traits enable them to survive longer, frequently blood feeding on humans, reproduce/breed, acquire and transmit *Plasmodium* sporozoite infections between humans even in the presence of high coverage of ITNs. For example, most of rural village of Kilombero Valley have 77.0% of ITNs coverage and 63.3% of ITN usage inside the house [86], which suggests that the area experiences residual malaria transmission. *An*. *funestus* prefer breeding in permanent and semi-permanent aquatic habitats (e.g., river streams, and ground pools/ponds). These large, permanent breeding habitats for *An*. *funestus* persist in both wet and dry seasons throughout the year relative to those temporary small and sunlit breeding habitats of *An*. *arabiensis* [87, 88]. Previous field studies demonstrated that *An*. *funestus* mediated more malaria transmission in different areas such as Tanzania [48], Burkina Faso [89], Benin [90], Cameroon [91], Malawi [92], Zambia [93], and Kenya [94, 95]. These studies linked the ability of *An*. *funestus* to dominate malaria transmission with their high sporozoite rates inside houses. However, some studies in other places have confirmed that both *An*. *funestus* and *An*. *arabiensis* may have similar high abundance or high sporozoite rates which indicates their equal contributions to malaria transmission [96]. Recent studies confirm that the population of *An*. *funestus* is increasing its dominance over *An*. *arabiensis* in terms of both abundance and sporozoite rates in certain African settings including Tanzania [57, 92, 97–99]. Furthermore, our study also observed a slight increase in abundance and sporozoite rates of *An*. *arabiensis* inside houses almost similar to the population of *An*. *arabiensis* in Zambia in an area with high coverage of ITN [100]. This observation suggests that *An*. *arabiensis* also increases their potential role in residual malaria transmission.

Malaria transmission intensity may be strongly linked with their blood feeding preference in humans (i.e. anthropophily) of malaria vectors. Our study revealed that both *An*. *funestus* and *An*. *arabiensis* obtained blood meal mainly from humans (high anthropophily) inside houses. While *An*. *funestus* fed on humans at a frequency of > 87.5%, *An*. *arabiensis* obtained human blood at a rate of 93% from inside houses in our study area [91]. Furthermore, our study also demonstrated that *An*. *funestus* and *An*. *arabiensis* can also flexibly blood feed on animals or mixed blood meal from animals and humans with *An*. *arabiensis* having highest degree of opportunistic behaviour than *An*. *funestus*. Such highest anthropophily behaviour in both *An*. *funestus* and *An*. *arabiensis* observed in our study area could be linked with the fact that mosquitoes collected from inside houses using CDC light traps and DN-mini traps were most likely fed on humans slept inside houses as their only accessible primary source of blood meal [77]. Both two species *An*. *funestus* and *An*. *arabiensis* seems to feed on human than on dogs indoors during wet season than dry season, this is consistent with previous observations of indoor feeding behaviour among the *An*. *funestus* and *An*. *arabiensis* [58]. Additionally, the feeding on humans by mosquitoes collected outdoors by both two methods DN-Mini trap and Prokopack aspirator across seasons, suggests that human hosts remain the primary target outdoors, despite availability of other potential hosts [53].The anthropophily in both vector species could also be linked with their high intensity of pyrethroid resistance that enabled them to contact ITN and continue blood feeding on humans inside houses in presence of ITNs [39, 101]. Additionally, the plasticity in blood feeding behaviour/mixed blood meals in both vector species could be associated with the common practice of most rural communities keeping dogs and chickens inside or near their human dwelling houses which increases chances of malaria vectors blood feeding on available alternative hosts [54, 102, 103]. Such mixed blood meals in mosquitoes are attributed with the low nutritional contents-quality/quantity of first blood meal ingested, host defensive behaviour (e.g. humans protected under ITNs), host movements (i.e. thwarting, skin shaking) and environmental factors (e.g. house modifications to mosquito-proof) which makes vectors to seek additional blood meal from alternative sources [71, 104–106]. Our findings agree with previous studies which demonstrate that *An*. *funestus* are highly anthrophilic in most African settings including Tanzania [54, 76, 92–95, 97], and they can also flexibly blood feed on alternative hosts such as dogs and cattle [53, 54, 92, 94, 96, 97]. Additionally, our result also agrees with other field studies which showed that *An*. *arabiensis* often exhibit opportunistic/plastic blood-feeding behaviour through obtaining blood from either humans or any available alternative host species such as cattle and dogs [48, 55, 56, 96, 100]. These previous studies suggest that the variations in blood-feeding behaviour patterns and host preferences of *An*. *funestus* and *An*. *arabiensis* may be linked with the availability and accessibility of their preferred host species such as humans and cattle in rural villages. Although *An*. *arabiensis* and *An*. *funestus* exhibited a certain degree of plasticity in their blood-feeding preference, they also expressed the highest anthropophily inside houses with ITNs which drives continued malaria transmission.

Our study also found that certain house improvements could reduce the risks of exposing humans to malaria-transmitting mosquitoes. Previous studies revealed that most of households in rural villages have open-eave gaps, unscreened ceilings, windows, and doors that provide the main mosquito entry points into the houses [107], and livestock such as cattle, goats, and chickens near their households which attract mosquitoes [53]. Our study found that while houses with metal roofing materials reduced the abundance of *An*. *funestus* inside house than those houses with grass roofing materials, houses with screened eave gaps reduced the abundance of *An*. *arabiensis* relative to those houses without screened eave gaps. Additionally, the houses with livestock had reduced the abundance of *An*. *funestu*s inside houses, but it never reduced the abundance of *An*. *arabiensis* inside houses. A possible explanation of our results could be that mosquitoes detect odour plumes emanating from human hosts to the outside via openings like eave gaps. Closing these gaps in houses prevents the flow of odour plumes to the outside of the house that guide mosquitoes towards house entry points. The lack of effect of livestock on abundance of *An*. *arabiensis* inside houses could be associated with their generalist host preference. These mosquitoes are equally attracted to and feed on both humans and animals based on host abundance and availability, and they can enter houses through various entry points. Our study supports those previous studies which found high numbers of *An*. *funestus* and *An*. *arabiensis* inside houses with grass/thatched roofing materials than those houses with metal roofing materials [26, 108]. These previous studies confirm that mosquitoes shift from resting on ceiling to other places including clothing, furniture, and floors due to unfavourable temperature and relative humidity that affect their survival inside houses with metal roofing materials [26]. Contrastingly, the houses with grass roofs provide optimal temperature, humidity environments for mosquito blood feeding, resting, egg production and survival. Also, our findings concur with previous studies which confirm that screened-eave gaps reduce the abundance of mosquitoes inside houses (e.g., *An*. *arabiensis* and *An*. *funestus*) compared to those houses with open-eave gaps [71, 109–112]. Although house improvement showed reduced vector density and risk of malaria inside houses, no protections were attained in some areas with low seasonal malaria transmission with high coverage of indoor-based interventions [113]. Furthermore, our study supports previous findings that keeping livestock near houses may reduce the abundance of malaria vectors inside houses (e.g., *An*. *arabiensis*, *An*. *funestus*) than those houses without animals, and it reduces mosquito biting rates on humans and risk of malaria [55, 65, 66, 113]. These previous studies further suggest that most malaria vectors including opportunistic *An*. *arabiensis* and anthropophilic vectors (e.g. *An*. *gambiae* s.s and *An*. *funestus*) are likely to be diverted to blood feed on cattle than humans, and rest inside/nearby cattle shed, which reduces mosquito biting rates on humans and sporozoite rates. Our study also supports those previous studies that demonstrated a similar density of *An*. *arabiensis* inside houses between houses with and without livestock [65]. On the contrary, other studies argue that keeping livestock like cattle close to houses may attract more mosquitoes to the houses that increase risks of mosquito entry inside houses, and mosquito biting rates in humans [53, 55, 63, 114]. Therefore, holistic house improvements (i.e. never single house components) and properly locating/positioning livestock houses/sheds could have a potential impact on malaria vector feeding preference, and risks of exposing humans to malaria.

The findings of this study suggest important implications for malaria transmission and effective complementary vector control strategies to ITN against residual malaria transmission. The increasing abundance, persistent anthropophily behaviour, and high sporozoite of *An*. *funestus* in areas with high coverage of ITNs suggest that vectors have evolved physiological resistance against ITNs that enable them to survive, blood feed on humans inside houses, reproduce, increase vector density and transmit malaria. For example, the population of *An*. *funestus* have exhibited high levels of *Plasmodium* sporozoite infections, and metabolic insecticide resistance against pyrethroids that enable them to sustain residual malaria transmission in most areas with high coverage of ITNs [39, 98, 115–117]. The improved ITNs including those integrating pyrethroids with Piperonyl Butoxide (ITN-PBO), Chlorfenapyr (ITN-Chlorfenapyr), or Pyriproxyfen (ITN-Pyriproxyfen) could be deployed to effectively counteract metabolic insecticide resistance in *An*. *funestus* and *An*. *arabiensis* in areas with sustained malaria transmission [22, 118, 119]. These vector species also expressed a certain degree of opportunistic blood feeding on livestock such as cattle and dogs. This vector behaviour enables them to obtain blood meal for their survival and reproduction leading to increased vector density while evading contact with ITNs inside houses to sustain residual malaria transmission. Accordingly, livestock-based interventions such as the treatment of livestock with endoctocides (e.g., ivermectin treatment of cattle–ITC technology) confirmed to reduce the survival and density of malaria vectors feeding on treated animal hosts [120–123]. Therefore, ITC technology could be an appropriate one health approach complementary to ITNs in the management of outdoor biting, animal feeding, and physiologically resistant vectors to reduce residual malaria transmission [23]. Our study also provided evidence that simple improvement of local houses through screened-eave gaps, metal roofs, and livestock husbandry reduces the density of malaria vectors inside houses. Because most houses in rural villages have open eaves, windows, and doors, and keep livestock which increases the abundance of malaria vectors inside houses, and mosquito biting rates on humans, the holistic house improvements, and proper positioning of livestock pens could be complementary vector control tool to ITNs in reducing the risk of residual malaria transmissions [22, 23, 124]. Overall, the improved ITNs, houses, and livestock-based interventions could be integrated to effectively reduce and eventually eliminate residual malaria transmission.

This study has also few limitations. First, the geographical scope of the study, it focused only on three villages of Kilombero Valley. This could restrict the applicability of the findings to other regions with different ecological and socio-economic conditions. Further studies on impact of house feature in vector bionomics could be performed in various ecological and socio-economical settings in the future. Second a significant proportion of blood fed *Anopheles* mosquitoes (i.e. 63%) were un-identified using ELISA. Such failure to detect host species specific blood proteins (antigens) could be linked to partial digestion of blood proteins (denaturation), or miss-match between antisera used in this assay and the specific proteins (antigen) for host species found in the study area. Future studies in our study area should consider using polymerase chain reactions (PCR) or proteomics such as Maldi-Tof techniques for blood meal analysis in mosquitoes.

## Conclusions

The current study demonstrates that variation in abundance, blood-feeding preferences, sporozoites rates of primary malaria vectors inside local houses, and their implications for selecting effective complementary vector control strategies to ITNs in Kilombero Valley. The higher abundance and sporozoite rates of *An*. *funestus* than *An*. *arabiensis* makes it dominating residual malaria transmission. However, these vector species continue to blood-feed on humans inside houses (anthropophily, endophily), and they can also opportunistically feed on alternative host species such as cattle or dogs in villages with high coverage of ITNs. Moreover, certain local house features significantly reduces the abundance of both vector species in rural villages. Therefore, a comprehensive intervention package that includes improved ITNs, holistic house improvements, and livestock-based measures (e.g., ivermectin treatment of cattle- ITC technology) could enhance the control of *An*. *funestus* and *An*. *arabiensis* that sustain residual malaria transmission in Kilombero Valley.

## Supporting information

S1 File Data are available within the supporting information files included in the submission portal. (XLS)

## List of abbreviations

CDC: Centre for Disease Control
CSP: Circumsporozoite protein
DN: Double Net
ELISA: Enzyme-linked immunosorbent assays
EMM: Estimated Marginal Means
GPS: Geographic Position System
GLMM: Generalized Linear mixed model
GTS: Global Technical Strategy
IRS: Indoor Residual Spray
ITNs: Insecticide Treated Nets
ITC: Ivermectin Treated Cattle
MUHAS: Muhimbili University of Health and Allied Sciences
NIMR: National Institute for Medical Research
PBO: Peperonyl Butoxide
PCR: Polymerase Chain Reaction
WHO: World Health Organization
